# Enzymatic Pre-Treatment Increases the Protein Bioaccessibility and Extractability in Dulse (*Palmaria palmata*)

**DOI:** 10.3390/md14110196

**Published:** 2016-10-26

**Authors:** Hanne K. Mæhre, Ida-Johanne Jensen, Karl-Erik Eilertsen

**Affiliations:** Faculty of Biosciences, Fisheries and Economics, Norwegian College of Fishery Science, UIT The Arctic University of Norway, N-9037 Tromsø, Norway; ida-johanne.jensen@uit.no (I.-J.J.); karl-erik.eilertsen@uit.no (K.-E.E.)

**Keywords:** *Palmaria palmata*, amino acids, protein, extraction, bioaccessibility, enzymatic treatment, gastrointestinal digestion

## Abstract

Several common protein extraction protocols have been applied on seaweeds, but extraction yields have been limited. The aims of this study were to further develop and optimize existing extraction protocols and to examine the effect of enzymatic pre-treatment on bioaccessibility and extractability of seaweed proteins. Enzymatic pre-treatment of seaweed samples resulted in a three-fold increase in amino acids available for extraction. Combining enzymatic pre-treatment with alkaline extraction resulted in a 1.6-fold increase in the protein extraction yield compared to a standard alkaline extraction protocol. A simulated in vitro gastrointestinal digestion model showed that enzymatic pre-treatment of seaweed increased the amount of amino acids available for intestinal absorption 3.2-fold. In conclusion, enzymatic pre-treatment of seaweeds is effective for increasing the amount of amino acids available for utilization and may thus be an effective means for increasing the utilization potential of seaweed proteins. However, both the enzymatic pre-treatment protocol and the protein extraction protocol need further optimization in order to obtain optimal cost-benefit and results from the in vitro gastrointestinal digestion model need to be confirmed in clinical models.

## 1. Introduction

Along with the expected world population growth in the coming decades, there will be a general increased demand for food, and particularly for proteins. Around 70% of the Earth is covered by water, but despite this, only 6.5% of the current global food protein consumption origins from the ocean, the main sources being fish and shellfish [1]. Besides fish and shellfish, there are many other marine species that could serve as valuable protein sources and among these are seaweeds. Seaweeds have long been a part of the diet in East Asia, but are not frequently used in other regions. The global production of seaweeds was around 25 million tons in 2012, of which 95% came from aquaculture with China and Indonesia as the main contributors [2]. In order to ensure a stable delivery of raw materials for industrial or nutritional purposes, cultivation is considered necessary.

Being plants, seaweeds are primary producers of macronutrients, such as carbohydrates, lipids and proteins. Elements like carbon, nitrogen and phosphorus are efficiently taken up from the environment into the cells and enzymatically converted to macronutrients, which are further used for growth or maintenance or stored intracellularly [3]. A large part of seaweed protein is thus situated intracellularly, in forms of newly formed amino acids or proteins, along with a wide range of enzymes. In order to optimize the commercial utilization of seaweed proteins, degrading the cell wall and liberating the intracellular proteins is of great importance. Seaweed cells are, like other plant cells and unlike animal cells, surrounded by a rigid cell wall functioning mainly as structural support and protection. The main constituents of the cell walls are complex polysaccharides, but also some proteins are embedded in it [4]. The cell wall polysaccharides are considered indigestible for humans, as the human gastrointestinal system does not contain the enzymes necessary for hydrolyzing the (1-4)-β-d-glycosidic bonds within them. In addition, they make ionic interactions with the attached proteins, hindering efficient extraction of these [5]. The protein bioaccessibility and extractability of seaweeds are thus lower than that of proteins of animal origin.

In a previous study [6], it was shown that several seaweed species are rich in proteins of good quality and thus that they could be utilized as protein sources in food and feed, or as ingredients in such [6]. Among the species in the mentioned paper, *Palmaria palmata* was found to be the best candidate for utilization in food and feed [6], and was thus chosen as a model species for this study. However, cultivation of this species has been shown to face some challenges when economic viability is concerned, and prior to commercial utilization of this species, these challenges have to be solved.

For protein extraction, several protocols have been developed, exploring the effects of osmotic shock, mechanical grinding, ultrasonic and enzymatic degradation of the cell walls [7,8]. The extraction yields have generally been low and also varied between studies, indicating that there is potential for optimization of these protocols. Concerning bioaccessibility, evaluated as the amount of amino acids available for absorption after gastrointestinal digestion, the literature is scarcer.

The aims of this study were thus to further develop and optimize existing extraction protocols and to examine the effect of enzymatic pre-treatment on bioaccessibility and extractability of *P. palmata* proteins. 

## 2. Results

The amino acid composition and protein content in untreated, homogenized and enzymatically treated *Palmaria palmata* is shown in Table 1. In the untreated samples, both total amino acid (TAA) content and the relative proportion of essential amino acids (EAA) were within the same ranges as previously described [6,9,10]. The amount of available TAA increased significantly both after homogenization and after enzymatic treatment with the polysaccharidases xylanase and cellulose, 1.7-fold and three-fold, respectively. Enzymatic treatment also increased the amount of available amino acids significantly compared to homogenization alone. However, there were no significant differences between the different enzyme concentrations.

**Figure 1:**
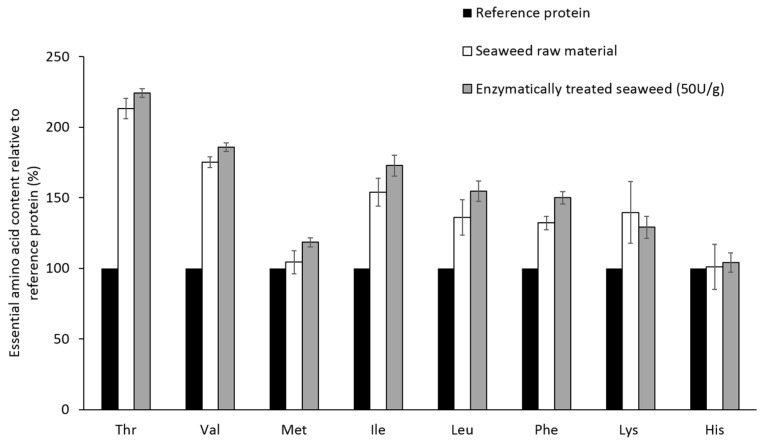
Essential amino acids composition (mg·EAA·g^−1^ protein) in raw and enzymatically treated *Palmaria palmata* relative to the reference protein set by WHO/FAO/UNU. The values are given as mean ± SD (n = 5) and in percent of the reference protein. Tryptophan is lacking due to destruction during acidic hydrolysis.

In Figure 1, it is shown that the amount of each essential amino acid in raw and enzymatically treated (50 U xylanase and cellulose g^−1^·alga) *P. palmata* proteins, is equal to or higher than the corresponding amount in the reference protein defined by FAO/WHO/UNU [11].

The results of the protein extraction experiment are shown in Table 2. Here, it is seen that alkaline extraction, either alone or in combination with 3.5% saline, was more efficient than 3.5% saline alone and ethanol in extracting alga proteins. Extraction at 60 °C seemed to increase the extraction efficiency compared to extraction at 23 °C, however, this effect was significant only for 0.05 M NaOH, 0.1 M NaOH and 0.1 M NaOH in 3.5% saline. Alkaline extraction following enzymatic pre-treatment increased the protein extraction yield significantly compared to all other extraction solvents and on both temperatures.

**Figure 2:**
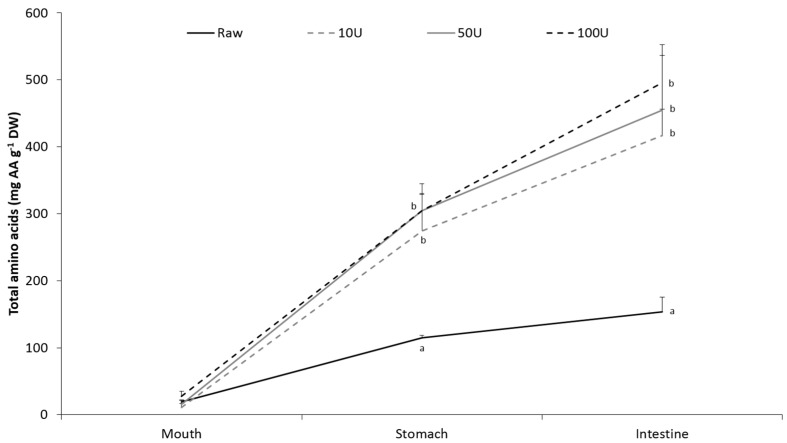
Total amino acids (AA) liberated in the mouth, stomach and intestinal fluids during gastrointestinal (GI) digestion of raw and enzymatically treated *Palmaria palmata*. Values are reported as mean ± SD (*n* = 5) and in mg·AA·g^−1^·DW of the source material. Different letters indicate significant differences (*p < 0.05*) between treatments, within each GI phase.

Following a simulated in vitro gastrointestinal digestion of raw and enzymatically treated *P. palmata*, the amount of amino acids available for absorption were significantly (*p < 0.05*) higher in all of the treated samples than in the raw sample (Figure 2). There were no significant differences between the different enzyme concentrations.

## 3. Discussion

Plant cells, including seaweeds, are surrounded by a rigid cell wall comprised of complex polysaccharides, with small amounts of proteins embedded in it [4]. Being primary producers of macronutrients, the algal cells contain large amounts of various enzymes involved in the conversion of absorbed elements to macronutrients. In addition, newly formed amino acids and proteins are stored intracellularly [3]. Extraction and subsequent utilization of these depend on disruption of the cell wall. In this study, cell wall disruption was performed using mechanical force, namely by Ultra Turrax homogenization, and enzymatic degradation. Cellulose is present in most plant cell walls. However, in red algae, the class in which *P. palmata* belong, xylans has been shown to make up a large proportion of the extracellular matrix, along with cellulose [12]. Thus, it was decided to use a combination of enzymes directed towards both of these polysaccharides for the experiments in this study.

Ensuring an adequate intake of EAA is necessary and when examining efficient protocols for increasing the amino acids available for hydrolysis it is important that the composition of EAA is not negatively altered. Previously [10], it has been shown that *P. palmata* proteins fulfill the demands of a complete protein, as defined by FAO/WHO/UNU [11]. This study confirmed the previous findings regarding protein quality (Figure 1) and the enzymatic treatment did not alter the EAA composition. Most common protein extraction protocols are based on the principle that cells burst due to osmotic shock when exposed to hypotonic conditions, and involve exposing the tissue to water or weak buffer solutions. This is a valid principle and an efficient procedure when extracting proteins of animal origin. In plants, however, the cell wall complicates protein extraction procedures. Plant cells hold a defense mechanism against osmotic variations, a mechanism in which intracellular vacuoles containing fluid of high ionic strength are central. When exposed to hypotonic solutions, water or buffer will flow into the vacuole, increasing its size and pushing the other cell organelles towards the cell wall. The intracellular pressure will thus increase, but the cell wall will prevent the cell from bursting [13]. Previous studies have shown that protein extraction protocols based solely on the osmotic shock principle are not very efficient for the extraction of seaweed proteins [5,14]. Several extraction protocols aiming at destruction of the cell wall, either by applying mechanical force or by enzymatic treatment, has been developed in order to overcome this problem [5,7,8]. The extraction yields have, however, been limited in most protocols.

In this study, several common extraction protocols were examined and modified in order to increase the protein extraction yield. It is well-known that the solubility of different proteins depends on the solvent used and in a previous paper it was shown that heat treatment increased the bioaccessibility of dulse proteins [10]. The extraction variables chosen were thus two extraction temperatures (23 °C and 60 °C), along with different types and concentrations of extraction solvents based on the solubility properties of different proteins. The solvents used were water, sodium hydroxide, sodium chloride and ethanol, along with combinations of these. Alkaline extraction following enzymatic pre-treatment was also included.

The extraction yields ranged from around 5% using 3.5% saline as extraction solvent at room temperature to 40% using 0.1 M NaOH as solvent at 60 °C (Table 2). Applying polysaccharidases for enzymatic destruction of the cell wall was shown to be more efficient than mechanical degradation, as extraction of the pre-treated alga resulted in an extraction yield of 75% at 60 °C, a 1.63-fold increase compared to the water-alkaline protocol (Table 2). This yield is markedly higher than reported in other studies using enzymatic degradation of the cell wall [5,8]. It is, however, difficult to compare results from different studies directly due to differences in the methods of protein determination, along with type and concentration of polysaccharidases used. 

After enzymatic treatment, the algae samples were subjected to a simulated in vitro gastrointestinal model (Figure 2) in order to investigate the effect of enzymatic treatment on the bioaccessibility of seaweed proteins. The liberation of amino acids into the digesta increased during the digestion process both for the raw samples and the samples exposed for enzymatic treatment. At the end of the process, simulating the end of the small intestine, the liberation of TAA was around 2.5–3.2 times higher in the enzymatically treated samples than in the raw samples. This increase corresponds well with the increased amount of amino acids available for digestion as a result of the enzymatic treatment as seen in Table 1 and indicates that GI digestion did not contribute to a further increase. The fact that the GI digestion was not more efficient in liberating amino acids from the enzymatically treated algae compared to the raw samples may indicate that the increased amount of amino acids released during the enzymatic treatment was not released as intact proteins, but rather as smaller peptides or free amino acids. As some of the intracellular proteins are non-specific hydrolytic enzymes normally participating in the cellular protein turnover, it is likely to believe that these may have contributed to a partial degradation of the intracellular proteins prior to the GI digestion. Around 4%–17% of the intracellular amino acids have also been shown not to be protein bound [3].

To sum up the findings in this study, it was shown that enzymatic pre-treatment of *P. palmata* increased the protein bioaccessibility and extractability, mainly by increasing the amount of amino acids available for hydrolysis. These results indicate that enzymatic pre-treatment of algae may increase the utilization potential of seaweed proteins. However, both the enzymatic pre-treatment protocol and the protein extraction protocol need to be optimized further in order to obtain optimal cost–benefit and results from the in vitro gastrointestinal digestion model need to be confirmed in clinical studies.

## 4. Experimental Section

### 4.1. Raw Material

Dehydrated *Palmaria palmata* was purchased from “Fremtidens mat” (Oslo, Norway). The seaweed was harvested at the south coast of Iceland. Following harvest, the seaweed was flushed with seawater and dehydrated at 40 °C for 24 h using electrical fans driven by geothermal energy. The dried seaweed was thereafter packed in airtight bags, before transport to Norway. Seaweed samples were cut into pieces of 0.5 cm × 0.5 cm prior to treatments. All chemical used in these experiments were of analytical grade and purchased from Sigma Chemical Co. (St. Louis, MO, USA) unless otherwise stated.

### 4.2. Water Content

Water content was determined using a modified version of AOAC method 950.46B [15]. Approximately 0.5 g of seaweed sample (*n* = 5), was dried at 105 °C until constant weight. Water content was determined gravimetrically. The water contents were only used for calculation of dry matter in the different fractions and results are thus presented as supplementary material (Table S1).

### 4.3. Protein Extraction

**Figure 3:**
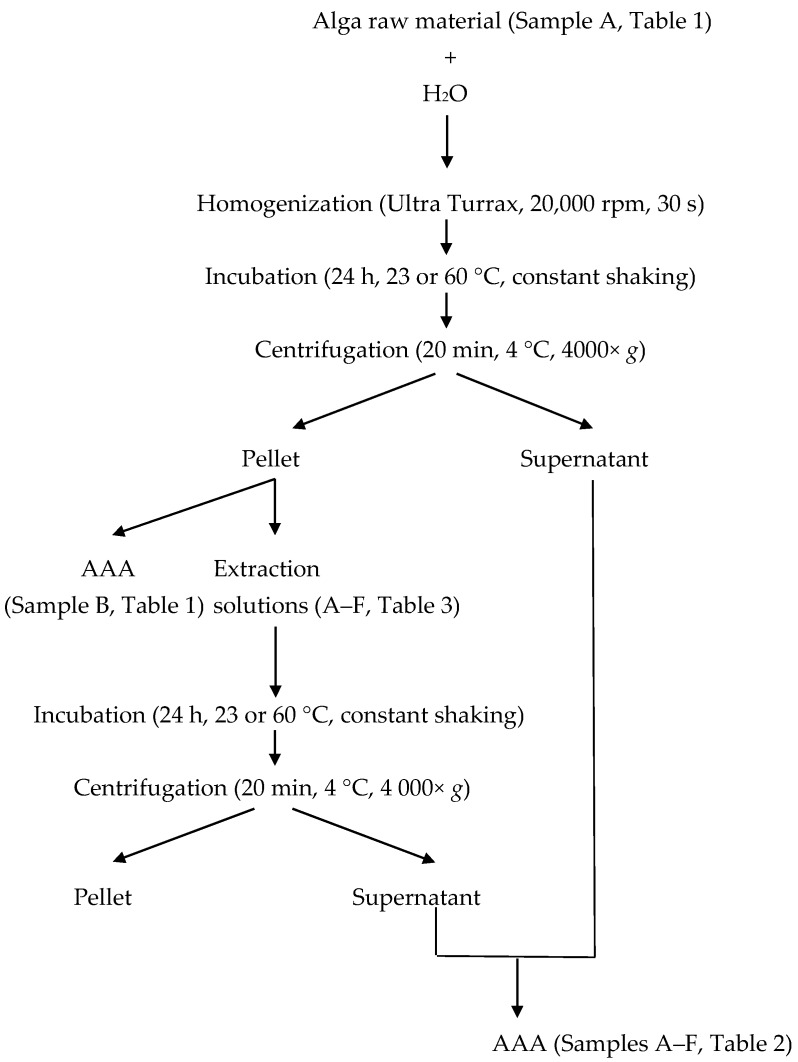
Flowchart of protein extraction and sample collections. AAA: amino acid analysis.

Protein extraction was performed according to Barbarino and Lourenço [7], with some modifications (Figure 3). In short, approximately 100 mg of milled seaweed samples were dissolved in 8 mL distilled water, homogenized using an Ultra Turrax T8 basic homogenizer (IKA Werke GmbH, Staufen, Germany) and incubated for 24 h at either 23 °C or 60 °C. The samples were centrifuged at 4000× *g* at 4 °C for 15 min. The supernatant was removed and the pellet was re-dissolved in 8 mL of the different solvents described in Table 3 and incubated for 24 h at 23 °C or 60 °C. The samples were exposed to constant shaking during both incubations. Samples were then centrifuged at 4000× *g* at 4 °C for 15 min. The two supernatants were combined and the final extracts were subjected to amino acid analysis.

### 4.4. Enzymatic Pre-Treatment

**Figure 4:**
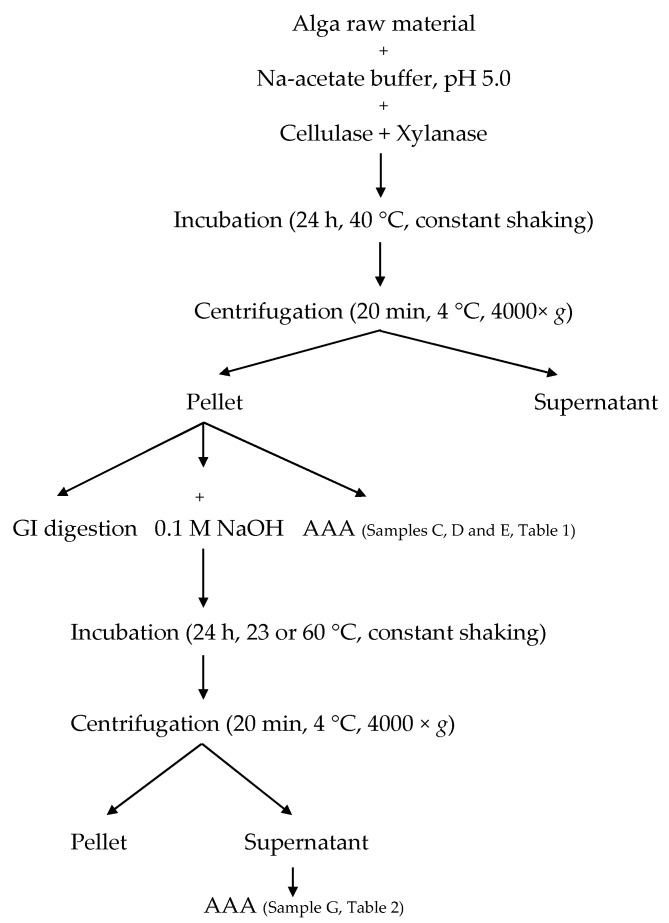
Flowchart of enzymatic pre-treatment and sample collections. GI: gastrointestinal, AAA: amino acid analysis.

Enzymatic pre-treatment was performed according to Harnedy and FitzGerald [8], with some modifications (Figure 4). Approximately one gram of seaweed was dissolved in 28 mL of 0.05 M sodium acetate buffer (pH 5.0), homogenized for 30 s using an Ultra Turrax T25 and incubated for 30 min at 40 °C under constant shaking. Enzyme solutions containing 10, 50 or 100 U xylanase and cellulase (both from *Trichoderma longibrachiatum*) in 2 mL sodium acetate buffer was added and incubation continued for 18 h at 40° C under constant agitation. Thereafter, the samples were centrifuged at 4000× *g* at 4 °C for 15 min, before separating supernatants and pellets. Pellets were subjected to amino acid analysis, in vitro gastrointestinal digestion and alkaline protein extraction. Algae samples without enzymes and buffer samples with enzymes were used as controls. 

For alkaline extraction following enzymatic pre-treatment, pellets were re-dissolved in 8 mL 0.1 M NaOH, incubated for 24 h either at 23 °C or 60 °C with constant shaking and centrifuged at 4000 × *g* at 4 °C for 15 min. Supernatants and pellets were separated and supernatants were subjected to amino acid analysis.

### 4.5. In Vitro Gastrointestinal Digestion

Raw seaweed and seaweed after enzymatic pre-treatment were subjected to a simulated in vitro gastrointestinal digestion model as described by Versantvoort et al. [16], with the modifications described by Maehre et al. [10]. Approximately 0.5 g of the seaweed samples were mixed with 6 mL of a solution mimicking salivary fluid (pH 6.80 ± 0.02) and homogenized with an Ultra Turrax T25 for 30 s, followed by incubation at 37 °C for 5 min under constant rotation. After centrifugation at 2750× *g* for 3 min, a 2 mL sample from the supernatant was collected and to the rest of the digesta, 12 mL of a solution mimicking gastric fluid (pH 1.30 ± 0.01) was added. The mixture was incubated at 37 °C for 120 min under constant rotation and the sampling procedure was repeated. Then, 12 mL of a solution mimicking duodenal fluid (pH 8.10 ± 0.02), 6 mL of bile solution (pH 8.22 ± 0.02) and 2 mL of 1 M NaHCO_3_ was added and another 120-min incubation at equal conditions was applied, followed by collection of a final 2 mL sample. For inactivation of the enzymes, all of the GI samples were kept at 90 °C for 5 min and then put on ice. Samples without seaweed were subjected to the same procedure and used for adjustment of amino acid contribution from the digestive enzymes.

### 4.6. Amino Acid Analysis

Raw and homogenized seaweed samples, along with pellets from enzymatic pre-treatment and supernatant samples from the different experiments were subjected to analysis of total amino acids (TAA). Sample preparations were similar to those described previously [10]. Approximately 200 mg of raw seaweed samples and pellets after enzymatic pre-treatment were dissolved in 0.7 mL distilled water, 0.5 mL 20 mM norleucine (internal standard) and 1.2 mL of 12 M hydrochloric acid (HCl). Samples were flushed in N_2_-gas for 15 s and hydrolyzed at 110 °C for 24 h, according to Moore and Stein [17]. Aliquots of 0.1 mL of the hydrolyzed samples were evaporated under N_2_ and re-dissolved in 1 mL lithium citrate buffer, pH 2.2. For the liquid samples (supernatants from all experiments and digesta from the GI model), 0.05 mL 20 mM norleucine and 0.55 mL 12 M HCl were added to 0.5 mL sample, before flushing with N_2_ and hydrolysis as described above. After hydrolysis, 0.1 mL sample was evaporated and re-dissolved in 0.5 mL lithium citrate buffer, pH 2.2.

All amino acid samples were analyzed chromatographically and identified as described previously [18] using a Biochrom 30 amino acid analyzer (Biochrom Co., Cambridge, UK). Tryptophan is destroyed during acidic hydrolysis and is thus not included in the results. 

### 4.7. Statistics

Statistical analysis was performed using SPSS 23 (SPSS Inc., Chicago, IL, USA). Tests of normality (Shapiro–Wilk’s test) and homogeneity of variance (Levene’s test) returned normal distribution with unequal variance for all chemical variables. Thus, one-way analysis of variance (ANOVA) was performed, followed by the Dunnett’s T3 post hoc test for evaluation of statistics. Means were considered significantly different at *p* < 0.05.

## Figures and Tables

**Table 1:** Amino acid composition in raw (A), homogenized (B) and enzymatically treated *Palmaria palmata* (C–E). The enzymes used were xylanase and cellulose in concentrations of 10 (C), 50 (D) and 100 (E) U·g^−1^·alga. Values are given as mean ± SD (*n* = 5) and in mg·AA·g^−1^·DW. Different letters indicate significant differences (*p <* 0.05) between treatments.

	A.	B.	C.	D.	E.
	Raw Material	After Homogenization	After Enzyme Pre-Treatment (10 U)	After Enzyme Pre-Treatment (50 U)	After Enzyme Pre-Treatment (100 U)
**Essential Amino Acids (EAA)**					
Threonine	8.9 ± 0.7 ^a^	16.8 ± 3.3 ^b^	25.7 ± 2.9 ^c^	30.4 ± 2.6 ^c^	27.1 ± 2.2 ^c^
Valine	12.4 ± 0.9 ^a^	22.6 ± 3.6 ^b^	36.9 ± 5.0 ^c^	42.9 ± 4.0 ^c^	38.5 ± 2.5 ^c^
Methionine	4.0 ± 0.4 ^a^	9.0 ± 1.7 ^b^	13.1 ± 2.2 ^b,c^	14.7 ± 1.3 ^c^	13.2 ± 0.8 ^c^
Isoleucine	8.2 ± 0.7 ^a^	15.8 ± 3.0 ^b^	26.2 ± 4.1 ^c^	30.0 ± 3.2 ^c^	26.8 ± 2.7 ^c^
Leucine	14.3 ± 1.8 ^a^	27.7 ± 4.7 ^b^	46.1 ± 6.3 ^c^	53.2 ± 6.0 ^c^	47.1 ± 4.8 ^c^
Phenylalanine	8.7 ± 0.6 ^a^	16.6 ± 2.9 ^b^	26.5 ± 3.0 ^c^	31.4 ± 2.4 ^c^	28.4 ± 1.6 ^c^
Lysine	11.0 ± 2.0 ^a^	19.5 ± 3.6 ^b^	28.8 ± 4.3 ^c^	33.7 ± 2.1 ^c^	29.5 ± 3.4 ^c^
Histidine	2.6 ± 0.5 ^a^	4.7 ± 0.7 ^b^	7.0 ± 0.8 ^c^	8.7 ± 0.5 ^c^	7.9 ± 0.8 ^c^
**Non-Essential Amino Acids (NEAA)**					
Aspartic acid *	21.9 ± 1.2 ^a^	32.1 ± 5.5 ^a^	50.9 ± 6.2 ^b^	59.2 ± 5.7 ^b^	52.8 ± 4.4 ^b^
Serine	10.5 ± 0.8 ^a^	20.5 ± 3.9 ^b^	31.8 ± 4.3 ^c^	37.7 ± 3.9 ^c^	33.2 ± 3.0 ^c^
Glutamic acid *	20.4 ± 1.8 ^a^	27.7 ± 5.6 ^a^	43.1 ± 5.3 ^b^	50.3 ± 5.2 ^b^	44.1 ± 3.1 ^b^
Proline	9.1 ± 0.4 ^a^	14.0 ± 3.1 ^a^	23.8 ± 2.2 ^b^	27.7 ± 3.8 ^b^	25.3 ± 2.4 ^b^
Glycine	12.1 ± 0.8 ^a^	20.7 ± 3.9 ^b^	32.4 ± 3.3 ^c^	37.6 ± 3.5 ^c^	34.6 ± 2.0 ^c^
Alanine	16.4 ± 1.4 ^a^	28.7 ± 5.2 ^b^	44.7 ± 7.0 ^c^	50.5 ± 5.1 ^c^	43.7 ± 3.1 ^c^
Cysteine	1.4 ± 0.4 ^a^	3.0 ± 0.8 ^a^	4.4 ± 1.7 ^b^	7.1 ± 1.2 ^b^	7.1 ± 1.4 ^b^
Tyrosine	6.9 ± 0.9 ^a^	13.3 ± 2.8 ^a^	23.6 ± 3.2 ^a,b^	29.2 ± 2.7 ^b^	26.2 ± 2.7 ^b^
Arginine	11.5 ± 1.1 ^a^	22.5 ± 4.4 ^b^	34.7 ± 4.9 ^c^	41.6 ± 2.3 ^c^	35.8 ± 3.6 ^c^
Sum	180.5 ± 12.3 ^a^	312.0 ± 54.2 ^b^	495.2 ± 59.5 ^c^	586.1 ± 53.5 ^c^	521.2 ± 40.7 ^c^
Relative amount EAA (%)	38.9 ± 0.6 ^a^	42.6 ± 0.9 ^b^	42.5 ± 1.2 ^b^	41.8 ± 0.3 ^b^	41.9 ± 0.5 ^b^

* Aspartic acid and glutamic acid represent the sums of aspartic acid + asparagine and glutamic acid + glutamine, respectively, as asparagine and glutamine are present in their acidic forms after acidic hydrolysis. Tryptophan is lacking due to destruction during acidic hydrolysi.

**Table 2:** Total amino acids and extraction yield in extracts of *Palmaria palmata* using solutions as described in Table 3, along with alkaline extraction following enzymatic pre-treatment (50 U·g^−1^·alga). Values are reported as mean ± SD (*n* = 5) and in mg·AA·g^−1^·DW for total amino acids and in percent of raw material DW for extraction yields. Different small letters indicate significant differences (*p* < 0.05) between extractions at 23 °C, while different capital letters indicate significant differences (*p* < 0.05) between extractions at 60 °C. * indicate significant differences (*p* < 0.05) between 23 °C and 60 °C using the same extraction solvent.

		Extraction Temperature			
		23 °C		60 °C	
	Solvent	Amount Extracted Amino Acids (mg·g^−1^·DW)	Extraction Yield (%)	Amount Extracted Amino Acids (mg·g^−1^·DW)	Extraction Yield (%)
A	0.01 M NaOH	55.8 ± 10.2 ^b^	17.9	59.9 ± 7.2 ^B^	19.2
B	0.05 M NaOH	80.6 ± 9.5 ^b,c^	25.8	118.1 ± 25.2 ^B,C,^*	37.9
C	0.1 M NaOH	90.1 ± 7.9 ^c^	28.9	122.0 ± 10.5 ^C,^*	39.1
D	3.5% NaCl	18.3 ± 4.7 ^a^	5.9	26.6 ± 7.0 ^A^	8.5
E	70% Ethanol	23.5 ± 4.6 ^a^	7.5	27.3 ± 4.6 ^A^	8.8
F	0.1 M NaOH in 3.5% NaCl	58.8 ± 13.3 ^b^	18.8	114.6 ± 19.2 ^C,^*	36.7
G	0.1 M NaOH following enzymatic pre-treatment	409.2 ± 46.0 ^d^	69.8	442.8 ± 86.5 ^D^	75.6

**Table 3:** Overview of the types and concentrations of the different extraction solvents used in this study, along with the types of extracted protein relevant for each solvent.

Extract	Extraction Solvent	Type of Extracted Protein
All	Water	Albumins
A	0.01 M NaOH	Glutelins
B	0.05 M NaOH	Glutelins
C	0.1 M NaOH	Glutelins
D	3.5% NaCl	Globulins
E	70% Ethanol	Prolamines
F	0.1 M NaOH in 3.5% NaCl	Combination
G	Enzymes + 0.1 M NaOH	Combination

## Supplementary Materials

The following are available online at www.mdpi.com/1660-3397/14/11/196/s1. Table S1: Water content of raw, homogenized and enzyme-treated *Palmaria palmata*. Values are presented as mean ± SD (*n* = 5) and in g·kg^−1^·alga.
