# Supplementary material for: Enzymatic Pre-Treatment Increases the Protein Bioaccessibility and Extractability in Dulse (Palmaria palmata)
Source: Mar Drugs. 2016 Oct 26;14(11):196. doi: 10.3390/md14110196 (PMC5128739; doi:10.3390/md14110196)
Supplement: Supplementary file 1 [file marinedrugs-14-00196-s001.docx]

Supplementary Materials: Enzymatic Pre-Treatment Increases the Protein Bioaccessibility and Extractability in Dulse (*Palmaria palmata*)

Hanne K. Mæhre, Ida-Johanne Jensen and Karl-Erik Eilertsen

**Table S1.** Water content of raw, homogenized and enzyme-treated *Palmaria palmata*. Values are presented as mean ± SD (*n* = 5) and in g·kg^−1^·alga.

|  | **Water Content (g·kg^−1^)** |
| --- | --- |
| Raw *Palmaria palmata* | 215.8 ± 15.0 |
| Homogenized *Palmaria palmata* | 908.1 ± 20.5 |
| Enzyme-treated *Palmaria palmata* (10 U) | 861.9 ± 27.1 |
| Enzyme-treated *Palmaria palmata* (50 U) | 864.2 ± 23.6 |
| Enzyme-treated *Palmaria palmata* (100 U) | 866.4 ± 15.0 |
